# “I feel it is not enough…” Health providers’ perspectives on services for victims of intimate partner violence in Malaysia

**DOI:** 10.1186/1472-6963-13-65

**Published:** 2013-02-18

**Authors:** Manuela Colombini, Susannah Mayhew, Siti Hawa Ali, Rashidah Shuib, Charlotte Watts

**Affiliations:** 1 Department of Global Health and Development, London School of Hygiene and Tropical Medicine, London, UK; 2 School of Health Sciences, Universiti Sains, Penang, Malaysia; 3 Women's Development Research Centre (KANITA), Universiti Sains, Penang, Malaysia

**Keywords:** Health providers, Intimate partner violence, Malaysia, Views and Attitudes

## Abstract

**Background:**

This study explores the views and attitudes of health providers in Malaysia towards intimate partner violence (IPV) and abused women and considers whether and how their views affect the provision or quality of services. The impact of provider attitudes on the provision of services for women experiencing violence is particularly important to understand since there is a need to ensure that these women are not re-victimised by the health sector, but are treated sensitively.

**Methods:**

In-depth interviews were conducted with 54 health care providers responsible for providing services to survivors of IPV and working in health care facilities in two Northern States in Malaysia. A thematic framework analysis method was employed to analyse the emerging themes. Interviews were coded and managed by using NVIVO (N7), a qualitative software package.

**Results:**

We found that when providers follow the traditional role of treating and solving IPV as “medical problem”, they tend to focus on the physical aspect of the injury, minimise the underlying cause of the problem and ignore emotional care for patients. Providers frequently felt under-trained and poorly supported in their role to help women beyond merely treating their physical injuries. What emerged from the findings is that time shortages may well impact on the ability of medical officers to identify cases of abuse, with some saying that time limitations made it more difficult to detect the real problem behind the injury. However, data from the interviews seem to suggest that time constraints may or may not end up resulting in limited care, depending on the individual interest of medical professionals on violence issues.

**Conclusions:**

Promoting empathetic health care provision is challenging. More awareness training and sensitisation could help, especially if courses focus on women’s needs and strengths and how health providers can validate these and contribute to a longer term process of change for victims of violence. Clear guidance on how to record history of abuse, ask questions sensitively and validate experiences is also important together with training on good communication skills such as listening and being empathetic.

## Background

Intimate partner violence (IPV) against women is a human rights violation and a global public health concern, as demonstrated by research over the past decade [1-3]. Globally, the WHO multi-country study on women’s health and domestic violence found that the lifetime prevalence of physical or sexual partner violence, or both, varied between 15% and 71% in 10 countries [4].

A major concern often argued in the provision of services for violence against women is to ensure that women are not re-victimised by the health sector, but are treated sensitively [5]. Many studies have discussed health providers’ views and beliefs on IPV and their impact on the quality of services [6-13]. Others have shown that their judgmental and directive attitudes and their lack of knowledge represent a major obstacle for abused women, often resulting in low disclosure [14-18]. Misconceptions and stereotypes around IPV are present among many clinicians, who may feel that violence is normal, who may hold negative views about providing services for IPV, or think that women are to blame for their husband’s aggression, [2,19-25], or who seem to be more focused on physical illness [7,26]. Health care providers’ clinical responses are shaped by own personal experiences and socio-cultural beliefs [27]. Providers often share the same cultural norms and practices of their patients, and similar gender values on IPV of the community – often experiencing violence themselves, as demonstrated by research from South Africa and the UK [7,22].

Moreover, the lack of training and knowledge about IPV combined with new tasks associated with IPV services have resulted in some health providers feeling overwhelmed and poorly equipped to intervene with abused women, and uncomfortable approaching IPV issues [9,23], especially when time is limited to deal with its social aspects [6,28-30].

### Responses to IPV in Malaysia

Health provider responses to IPV and their effect on service provision are context and situation specific. This article focuses on a Malaysian model of health response to violence against women called One-Stop Crisis Centre (OSCC).

The OSCC model has been implemented in emergency departments of regional, specialized and district public hospitals (with various degrees of differences) by the Ministry of Health to offer medical and health services to domestic and sexual violence. At the OSCCs, women who experienced domestic violence receive on-site medical, psychological, and social support [31]. The first OSCC was established in 1994 at the General Hospital, Kuala Lumpur, in collaboration with women's non-governmental organizations (NGOs), and was located within the Accident and Emergency (A&E) Department. Soon after, the Malaysian OSCC became a unique integrated model of care to women experiencing abuse, being implemented nationally through public hospitals across the entire country [32]. Subsequently, other countries in the South East Asian region also replicated this model [33,34].

Only a few studies have been published in Malaysia about providers’ views and attitudes towards violence against women in general, mostly at primary health care levels [35-38]. For instance, a study to assess the knowledge, attitudes and practices of primary health care providers regarding the identification and management of domestic violence in a hospital setting in Malaysia found that 28% of the clinicians and 51.1% of the nursing staff had inappropriate personal values regarding domestic violence and blamed the women for having done something to trigger their partner’s reaction [37]. Moreover, the same study shows an underlying belief that domestic abuse is a rare and 'private matter'. More than half of the clinicians and a third of the nursing staff reported a fear of offending patients in asking about abuse. Over sixty percent of health care providers believed that the prevalence of domestic violence among their patients to be low [37].

However, these studies do not fully reflect the range of attitudinal and systems challenges health workers are facing when trying to integrate a new package of services for women experiencing abuse. This article aims to fill this gap by exploring the views and attitudes of health providers towards IPV and abused women, and to investigate their impact on the provision and the quality of OSCC integrated services in Malaysia.

## Methods

We employed a case study approach in this qualitative descriptive study allowing in-depth analysis of seven hospitals in 2 states in Malaysia. The aim was to analyse barriers and opportunities to implement and integrate effective health service responses to IPV at different levels of the health system in two Northern States in Malaysia. In particular, the overall study sought to: 1) explore and understand providers’ attitudes towards violence, 2) assess the training and organisational support they might receive to implement the policy around IPV, and 3) the challenges they faced when offering IPV services. The findings on policy developments and health systems issues have been published elsewhere [39,40]; this article presents the results from the health staff interviews on their views and opinions towards abuse and abused women (definition, acceptability, and their role as service providers) and how these can affect service provision.

In-depth interviews were selected as they can provide reliable and comparable data [41], and allow for control over the questions and the topics to be covered in the discussion while leaving the interviewee the opportunity to tell his/her own account of issues related to the selected topics [3]. Between January to April 2007, a total of 54 in-depth interviews were conducted with selected health providers (including nurses, medical officers, gynaecologists, medical social workers and hospital managers) who were responsible for providing services to abused women at different levels of hospital care: tertiary specialised facilities (Regional specialised hospitals), secondary ones (semi-specialised hospitals) and basic district settings (basic facilities with no specialists on site). Table 1 provides a summary of the type of providers interviewed.

**Table 1:** Numbers of interviews per type of providers

**Respondents**	**Penang**	**Kelantan**	**Total**
***Counsellors***	1	1	2
***Medical assistants***	0	2	2
***Medical officers***	14	8	22
***Medical Social Workers***	3	2	5
***Obstetrics and Gynaecologists (OBGYN)***	3	2	5
***Psychiatrists***	2	2	4
***Staff Nurse/Nurse***	8	6	14
***Total***	**31**	**23**	**54**

Staff were interviewed at a total of seven hospitals selected in two Northern States. In each State, a tertiary specialised hospital was selected, together with a secondary and a district setting (to get a sample of different levels of service with an OSCC). Snowball sampling was used to identify health providers, with the assistance of the local partners from the Universiti Sains Malaysia and the Head of A&E Departments. Respondents were selected according to their profession and their experience with violence issues and their connection with OSCCs.

Semi-structured interview guides were developed – and subsequently field- tested - to offer core questions around providers’ views about violence and their challenges when providing services to abused women. Key topics contained in the interview guides were:

– Perceptions and views around domestic violence, abused women and their role as providers of IPV care: to understand whether providers’ attitudes impact on the services provided.

– Clinical management: to help map the process of OSCC care and the daily practices around clinical management of abuse cases.

– Guidelines and protocols: to get a sense of providers’ awareness and knowledge of hospital’s protocols and procedures around violence.

– Training: to get a sense of knowledge of IPV that providers may have.

– Collaboration with other agencies: to understand whether processes of collaboration among various sectors exist and how it works, and also providers views about it.

– Perceptions of challenges faced when providing IPV services: to understand personal opinion about perceived barriers/challenges when dealing with abused women.

Written informed consent was obtained from each respondent. The majority of interviews were conducted in English by the principal investigator. Twelve interviews (primarily with nurses) were conducted in Bahasa Malaysia by two local research assistants, who had been previously trained on conducting qualitative interviews and on the content of the interviews-topic guides. The average length of each interview was around 45 minutes.

Digitally recorded interviews were saved onto a computer and subsequently transcribed. The twelve Malay interviews were subsequently translated verbatim into English. A local person and the field supervisor in Kelantan checked their fidelity towards the Malay version. A framework analysis method was then employed to analyse the emerging themes [3,42]. It consists of a content analysis method allowing for a systematic classification and organisation of data by major themes, categories and concepts within a thematic framework [3]. This approach encourages the preservation and integrity of the voices and accounts of the interviewees, keeping the researcher grounded in the data, as the information is summarised and classified within a thematic matrix all along the analysis [3].

The transcripts of in-depth interviews were read repeatedly to become familiar with the text, to have a full picture of the data collected, and to begin to identify some main themes throughout such initial reading. Once the cross-cutting thematic code framework was finalised, interviews were coded and managed by using NVIVO (N7), a qualitative software package.

Throughout the analysis, the code framework was further revised where new sub-codes and themes were identified and as linked themes were grouped together, reducing the number of broad overarching issues.

Ethical approval was granted by the Ethics Committees of the LSHTM and the World Health Organisation. Ethical permission for the study was also granted by the Economic Planning Unit of the Prime Minister’s Office and the Ministry of Health national ethical review committee in Malaysia.

## Results

Fifty-four in-depth interviews were conducted with health providers (nurses and medical officers) from seven public hospitals (regional and district settings) in two Northern States in Malaysia (see Table 1). We explored how IPV and abused women were perceived by health care providers and the main effects of this on the perceptions of their role as providers. We then looked at the challenges faced by service providers when offering care to abused women.

### Health providers views and perceptions concerning IPV

Over half of the respondents defined IPV as both physical and emotional harm caused to the woman, and included acts such as scolding, hitting, beating, threats, emotional stress and deprivation.

“*It doesn’t have to be physical, it could be emotionally and not just physically, just…some people would just abuse whoever women in their life, either physically hurt them but could be emotionally as well. That is considered abuse for me…like if you feel threatened by somebody or if you feel uncomfortable by somebody I think this is considered violence…I mean if you are violated in some ways.”* (Pen/45, OBGYN, female)

Very few providers perceived IPV as hitting and physical abuse only.

“*Mainly I think now, it is more on physical rather than those things* [emotional deprivations]*…”* (Pen/3, medical officer, female)

When talking about physical abuse, one medical officer said that many health professionals still refer to it as “just” a social problem, and not as a criminal offence, unlike child or sexual abuse. Moreover, he added that some male medical professionals seem to judge it more as a “social problem” if abused women presented with minor injuries, which may reflect the A&E goal of responding to emergency situations. Therefore, urgency among some medical personnel seems to be related more to the severity of the physical injury.

Only a quarter of the providers in Penang mentioned sexual abuse among the types of acts that may characterise domestic violence. One medical respondent clearly stated that rape was not a form of family violence.

“I: Would it also be sexual? Would also be considered?

*R: Sexual abuse, rape, I don’t think, sexual abuse no.”* (Kel/11, medical officer, female)*.*

This may reflect the legal dimension of domestic violence in Malaysia, which (at the time) did not include marital rape in its definition.

Some minor discrepancies in the overall definition of domestic violence existed between providers’ views in the two States, but there did not appear to be marked differences based on the sex of the health professionals. In Penang, most people referred to IPV as causing both physical and psychological harm, unlike in Kelantan, where respondents referred primarily to hitting and beating and any physical injury. These differences may be caused by the more limited referral resources in Kelantan State or by the religious context.

#### Attitudes towards acceptability of IPV

Over three quarters of the providers interviewed thought IPV was something that should not be tolerated. The majority of all the health care respondents said that no form of violence by the husband or partner was acceptable or used as a tool to solve arguments among couples.

*“I think it is unfair, unfair. It is unfair, it is illegal, I mean things can be done because we are both humans and uh, being a family means a family shares everything. Not, nobody is higher or lower than the other one*.”(Kel/10, medical officer, female)

One staff nurse even said that offending husbands should be punished.

*“If a husband beats his wife,. I think he should be put behind bars…I guess police have to take action against her husband for treating his wife like that, take a legal action.”*(Pen/13, staff nurse, female)

Despite generally intolerant views towards IPV, there were still a small minority of medical officers and staff nurses at district and more specialised hospitals who accepted it or minimised it, referring to physical abuse (such as a slap) as a “small thing”. However, even among the very few providers who reported they would condone minor occasional physical abuse, one medical officer stated that if the violence was severe and frequent, he thought it should be tackled.

*“If it happens only once, we may be able to compromise. If it has happened many times and has left behind a deep effect (on the victim), such as excessive injury marks on the victim’s body, I would suggest that the wife be divorced from her husband, for her safety and peace of mind. We can forgive the husband if the abuse is the first time. When the abuse happens for the second time, we can still forgive the husband. The abuse could be unintentional or the husband is under a certain pressure. But if the abuse happens frequently, I think it is no more a peaceful home but a hell for the victim.”* (Kel/16, medical social worker, male)

Among a minority of providers who accepted violence as a normal part of married life, a nurse offered a different kind of advice to women. She would tell them to be patient and stay with the husband - because of the children - or advise them not to divorce.

“I: so what do you tell them to calm them?

*R: (laughs) what did I tell? I advise them to be patient, that’s all. [.].I mean, you be patient, you have to think about your children [.] So we just say sabar (patience). What to do, isn’t it? Just sabar (patience). Be calm.”* (Pen/19, staff nurse, female)

Over half of the medical doctors and staff nurses viewed women as passive and powerless actors accepting or tolerating abuse for many years. According to nearly half of all the providers, women hid the abuse and were often not telling the truth, did not want to disclose or report violence because of fear of repercussions, because they wanted to save their marriage, or due to cultural reasons. This image may be a function of the perception of the community about violence as culturally tolerated within a family – but publicly unaccepted – and to be kept hidden.

The majority reported that most of the time women tolerated the abuse and continued to bear it for many years because they were not aware that marital violence was wrong.

“*Because it’s probably awareness. They do not know probably even know that it’s wrong to do violence against them. Awareness I think. They do not know it is wrong. To not know it’s wrong, you do not complain, they just accept it.”* (Pen/45, OBGYN, female)

This could be due to the widespread silence and denial surrounding IPV among the community.

“[.] They do not even know that it’s wrong to do violence against them. [.] They do not know it is wrong. To not know it’s wrong, you do not complain, they just accept it.” (Pen/45, OBGYN, female)

Four doctors reported that women who were in abusive relationships saw no other way out than staying.

*“I think the majority, they are stuck in an abusive relationship. Especially the women can’t see a way out I think, just carry on with the abusive relationship.”* (Pen/4, medical officer, male)

Others stated that some women would blame themselves for the abuse, thinking “*that they deserve to be treated that way.”* (Pen/27, counselor, female)

### Perceptions of services: expectations and roles

#### Providers’ views on women’s expectations from the OSCC services

To explore their perceptions of women’s needs and whether these may impact on the services provided, practitioners were asked about their views on women’s expectations from OSCC services. When asked what women expected from them, a range of different perspectives were given. Nearly half of the providers reported that women just wanted medicine and to get medical treatment and a diagnosis of whether their injury was serious, and did not want any counselling or referral.

*“They just want to make sure that they’re alright…. very few of them would want to be counselled.”* (Pen/20, medical officer, female)

On the other hand, several respondents (both clinical and non clinical staff) also felt that some women went to OSCC to receive counselling and advice from the health staff to help them solve their problem, and that they did not solely come because of the injury.

*“To help them, what they expect actually is to help them. […]… they don’t come because of their pain, err… they come because of their problems. like domestic violence,. probably they want us to help…”* (Pen/42, medical officer, female)

Few clinicians admitted that women used hospitals as their first entry point for a “help call”, even when they present with minor injuries.

*“.sometimes they do not know where to go, so that’s why they come to see doctor, the injury is not that severe but they don’t know where to go…”* (Pen/42, medical officer, female)

More than ten providers stated that some women came to the services because they wanted to obtain a medical report to get a proof of the abuse and their suffering in order to make a police report for a divorce or take other legal action against their husband.

#### Providers’ perception of their role as “health care workers”

Providers were asked about the perception of their role as “health care workers” in order to explore whether their view would affect the provision of OSCC services. What emerged was that health professionals viewed their role as varying between a purely medical one – focusing primarily on offering treatment and diagnosis – to a broader role as “advisers” guiding abused women and channelling them to proper services to tackle the underlying causes.

Some differences in response existed across levels of hospital care. At the tertiary level, several providers reported they had a duty to reassure women and offered them “options” about available services.

*“[.] It is the duty of the medical officer to inform them of these options because they will not go directly to the women crisis centre, unless they are very educated. The non-educated patients will not know that this crisis centre exists.[.] But they know that there are doctors in the hospital that they can seek treatment from. [.] I think that is the role of the medical officers.”* (Pen/3, medical officer, female)

Helping women solve their problem was a common explanation of their professional role among various health providers at higher level of care. Help could be offered in the form of advice on support services, reassurance and by referring women. The majority of providers at the tertiary level stated they would provide further support to women by assisting them beyond treatment by referring them to other departments, though noting that hospitals could only focus primarily on physical and counselling aspects.

*“…as a medical officer, this is the way I can help them… try to calm them, try to see that it is not the end of life yet and we have the way to solve their problem, and try to refer them to the respective persons who are able to solve their problem… Hospital mainly they can concentrate on physical and counselling aspect only. ”*(Kel/6, medical officer, male)

Many also reported that they would ensure women felt comfortable, and reassured them that there was someone to help them. However, one doctor mentioned that their role was limited because they could only make suggestions about where to seek help because they were too busy.

*“So we will try to offer whatever we have… we are giving them some suggestions where they can find solutions. That’s the only thing we can do, more than that I don’t… because we* [A&E] *are a busy setting…”* (Pen/2, medical officer, female)

Many clinicians seemed to show empathy towards women, especially at tertiary level. For instance, thirteen reported they would go slowly with them, lower their voice, close the curtains or clear the examination area to make women more comfortable. They would talk slowly, minimise the examination discomfort and listen sympathetically. On the other hand, at district hospitals, the main focus of health personnel seemed to be more on physical examination treatment.

*“[.] Actually, for us it is physical treatment only, [for] the social problem, social welfare is supposed to take over…”* (Pen/12, medical officer, male)

Some of those who stated that their role was focussed on the medical aspect of IPV also reported that the social part was done by the police.

*“…we let the police take over the case. Why it happened, what causes, we don’t bother about that. [.] No further history about the event, the causes. There we need to let the police take over about that…we really concentrated at the physical examination and do diagnosis… we are not interested* [in the causes] *because we are also not counsellors.”* (Kel/12, medical officer, male)

### Challenges of service provision

#### Frustrations reported by providers

Nearly half of the health professionals at all levels expressed at least some frustrations because of their feeling of inadequacy, as they could not assist women properly. In particular, at district hospitals, some confessed their frustration for their inability to help women solve their problems beyond medical treatment.

*“….what I am doing now, I feel it is not enough because I’m just doing the basic counselling, identify their problem and referring them to the other units, you see. […] most of them won’t come back but I feel very depressed because I can’t do much.”* (Pen/7, staff nurse, female)

Some doctors at tertiary level also reported feelings of inadequacy, as if IPV was beyond their reach and should be dealt with by psychologists or social workers. The problem was raised particularly when there was a need for long-term care to women such as solving marriage and family problems, which they stated were not included in their traditional medical role. Some said they could only offer short-term help for physical and emotional abuse, but they could not impact on any longer-term problems.

At least a quarter of the providers suggested that health personnel’s anxiety was the result of the lack of any training or awareness on how to manage cases properly and the lack of basic counselling skills (e.g. how to offer advice to women or ask questions sensitively). This was particularly evident at district level, where no specialists are available and where several providers reported they felt women with minor injuries were more in need of counselling and someone to talk to than of medical help.

At district level particularly, it seemed that providers’ perceptions of their role was influenced by the level of resources available in their settings. For instance, some reported that they felt their role was limited to treatment, medication and referral to further specialised support, as there was very little they could do at district level apart from listening and offering their advice to women. In this case, their role seemed limited not by their unwillingness to help or by their view of their role as purely medical, but more by the availability of resources.

*“There’s very little we can do. That we can only reassure them that there’s nothing wrong, medication, tell them we’re around here if they need anything.”* (Pen/18, medical officer, female)

Despite being committed and empathetic, nearly half of all providers - when talking about their professional role - reported some frustrations whilst dealing with abused women. Some workers reported feeling helpless because they could not force women to receive assistance. If women were not willing to be assisted or advised, providers felt they could not do much. For instance, some reported they got frustrated because women did not want to report to the police or be referred to specialists. Some of the reasons stated were related to women wanting to save the marriage (because they love their husband), to protect the children or because they do not have any other income besides the husband.

Frustration also seemed to be common among all providers when women did not disclose and were hiding the truth, making it more difficult for health care workers to identify and address the problem. The low disclosure was attributed to various causes. Some health care workers at tertiary and secondary level hospitals attributed the unwillingness to disclose among abused women to the local culture, reflecting the general view about IPV as a family private issue.

At least ten providers reported frustration with repeated cases, when women kept coming back to hospital with the “same” problems.

*“Once they come, after treatment, they go back to their old instincts, and then relax and they come back again. Similarly….when the patient came in, we gave treatment and back and they come again for the same mistakes. It happens again and again and the similar problems will be coming in A&E. That’s what we feel.…”* (Kel/13, medical assistant, male)

Several providers also stated they felt frustrated with feeding information to women and not knowing whether women may accept their advice on referrals or not. Others also reported they could not understand women’s decisions to remain in abusive relationships, despite the abuse, and felt frustrated because they could not influence their decisions.

*“Sometimes they* [providers] *feel very frustrated because we try to help them [women] from being abused, then they go back to the same person and get abused again. It’s very frustrating…”* (Kel/7, OBGYN, female)

Only two respondents seemed to recognise the courage that it takes for a woman to disclose the abuse and seek help.

#### Time pressure

Over thirty providers cited time pressure as an important constraint to providing quality OSCC care. Doctors mentioned that time pressures were a constraint, as they examined women, and short examination time was a particularly common issue at lower levels of care, where some doctors said they were busy with other patients. These pressures were not reported by nurses although time constraints were said to be an embedded problem of the frenetic environment of A&E as other emergency cases kept coming in all the time.

*[…] here most of the time is very busy (laughing) we cannot really concentrate on them [abuse cases] actually, we just treat them as usual patient, just take history, examine and then go back just like that la…* (Pen/44, medical officer, female)

What emerged from the findings is that time shortages may well impact on the ability of medical officers to identify cases of abuse, with some saying that time limitations made it more difficult to detect the real problem behind the injury - especially when women did not disclose. It was also cited as a barrier to asking the proper questions to assess women’s emotional needs for further referral.

“*…we don’t have enough time, because a lot of cases… the outpatient cases after office hours also come here, so we don’t have enough time to go in the separate room, to take a long history, so what usually happens, we are not going to ask the reasons why she was battered and go in deep depth on that. We just ask about what time, place, what weapon or thing that was used to hit the woman, do the physical examination then ask whether they are willing [to have] some counselling or refer…”* (Pen/6, medical officer, male)

Nevertheless, data from the interviews seem also to suggest that the individual interest of medical professionals on violence issues may influence whether or not time constraints result in limited care. For instance, some doctors stated that if the physical injury was minor, and if they saw IPV as just a physical problem, they might spend very little time with such cases.

“*[…] sometimes they* [doctors] *have cases, we have the room available, but they prefer to see the case here. They want to see it very fast, for them is to see the injury…”* (Pen/7, medical officer, female)

At a secondary care level, it was also a matter of some providers being committed to “go the extra mile” and be interested in the woman’s story. A gynaecologist reported that most of the time, doctors would just ask very few questions and then go over to the next patient.

*“Most of the time we don’t give that much time. We just go to these patients, ok, ask them a few questions… and not willing to talk anymore, but you don’t go the extra miles. I mean, if you put them in a room somewhere and ask them more questions they probably want to tell you more. But sometimes it’s just ‘ok, next patient… next patient’.”* (Pen/45, OBGYN, female)

At other times, clinicians reported that they might not feel “comfortable” to spend a long time with women, especially when they would not disclose and would remain silent.

*“We don’t give the* [women] *that much time. We just ask “ok, what happened? Ah ah”. Because there will be some women and there will just be silent and do not know what to say to you… sometimes we don’t give them that time… ok, silence now, [we] are not comfortable now, better ask another question, time’s out now… we jump to another question, so that would be the problem sometimes.”* (Pen/45, OBGYN, female)

The discomfort expressed by some providers in asking women about more “personal” questions may prevent them from identifying the real problem behind the abuse. Some providers felt ill equipped to talk to women, to ask them the proper questions about the abuse, and thus they may use shortage of time as an excuse to have a quick consultation and move on to the next patient. Some also saw the process as merely a clinical task, therefore they may be unable to focus on more personal questions during the “examination process”.

## Discussion

Most health care providers interviewed defined IPV as physical and emotional abuse only, and included acts such as scolding, hitting, beating, threats, emotional stress and deprivation. Only a few providers mentioned sexual abuse among the types of acts that may characterise IPV. There seemed to be some hesitation in defining IPV, as if the providers never thought of it before. This could reflect the lack of any reflective training when the OSCC services were integrated.

Our study also shows that there is a predominant perception of IPV as a family or marital issue, and therefore IPV is not seen as a public health matter, despite the fact that some acknowledged the physical and emotional consequences resulting from it. In general, IPV is not perceived as a priority health issue, probably, as some doctors said, because it is not “life threatening”. Although many disagree with violence as a means to solve marital conflict and label it unfair, being part of a culture that closes its eyes to violence against women seems to impact on the views of some health care professionals, and therefore may fall down the list of priorities in a busy A&E context, where most of the abuse cases may be found.

The findings presented in this article support the view that providers’ attitudes to IPV and their perception of their role could affect the quality of the services they offer. Our interviews showed that there seemed to be a link between providers’ view of IPV and of their perceived role and how they therefore responded to survivors of IPV. Providers who demonstrated less understanding of the socio-cultural determinants of abuse were also the ones who were more likely to focus on the injuries. For those who were more focused on the physical consequences of IPV, the emotional part of the care was frequently disregarded or not seen as part of their role. Thus if providers thought that their role was just within the medical domain, they might fail to recognise the complex interaction between medical and psycho-social aspects of IPV care, and were less likely to refer women for any additional services they may need. This reflects the findings of another study which shows that the psycho-social aspects of medical care are often undervalued, where the emotional aspect of IPV care goes often ignored and its ‘*social emergency’* is unrecognised [43]. This lack of awareness of the psycho-social dimensions of IPV risks perpetuating the view that IPV is purely about a couple quarrelling which in turn can lead to inappropriate responses that could jeopardise women’s safety and impact on women’s views of the services as they can lose trust in the provider.

The study findings show that many providers could not empathise with women’s decisions of going back to their husbands, and women’s evasiveness and underreporting were often causes of frustration for them. Their sense of medical responsibility towards abused women and the perception of their role as “solver of patients’ problems” might have limited their empathy towards women – especially the ones who chose to remain in abusive relationships and do not accept their advice. In fact, despite their willingness to help, some still lacked respect for women’s choices. This may arise more from lack of understanding about how difficult it is for women to disclose or to leave an abusive relationship, especially without specialist support (emotional, practical, legal etc.), and also derives from low understanding about IPV being characterised by power and control issues. For example the existing literature [9,23] found that health professionals often do not comprehend that some women do not really have any alternatives, and often feel incapable of trying to influence patients to report, seek additional care and in referring them appropriately.

Our data show that some doctors see women as an obstacle in their perceived self-efficacy in the management of IPV, and do not understand the barriers women may face such as their financial and legal dependency on their husband, or the blame of the community, and the shame of a divorce. Not all respondents really recognised the courage required from women when seeking help. Such an attitude could be linked to the way some providers tend to focus on “fixing” the medical aspect of abuse and would tell a woman to leave the husband, rather than thinking about what the woman may really want. This may reflect the medical culture, which is primarily curative and thus sees the provider as the main decision-maker [44]. This behaviour may also be related to a social class divide between doctors, who often belong to a higher socio-economic class than women who experienced abused. Moreover, this feeling of frustration, especially with “uncooperative women”, could be linked to the fact that providers think that it is difficult to offer an effective solution to women, especially when “successful” means convincing them to leave their husband. This issue is also raised in other settings [7]. Providers may lack understanding of women’s disempowerment – and the social context of abuse and the gender inequalities leading to IPV - due to their social distance from the community, and thus their lack of understanding of the problem. Moreover, there was no understanding that their role may be feeding in a longer term process of change among abused women. Even if a woman does not feel able to leave a violent relationship, she would like recognition and support from her health provider, without being pressured to any action. This issue has been explored in other industrialised countries [16]. Some women may not be ready to leave their husband and if health providers do not understand it, they may place women at more risk. More patient-centred and “stage-matched” interventions could be elaborated according to the various stages of change with regard to abuse [17]. Health professionals should at least support women’s decisions, and, in the long run, contribute to the women’s ability in making a change to their situation [45]. Referral to community support groups or NGOs, where they exist, can also be done and has been quite successful for example in Uganda (Michau and Nakar 2003).

There is the ongoing question about health care workers’ roles and in what ways they should be expected to help women. The contentious issue is whether their role should be purely medical or go beyond treatment [27,46]. The holistic management of IPV is not universally accepted as part of health personnel’s medical routine [7,47]. Our study shows that when providers follow the traditional role of treating and solving IPV as a “medical problem”, they tend to focus on the physical aspect of the injury and minimise the underlying cause of the problem. This does ensure that they at least treat the physical injuries of the patients, but it risks detaching providers from women’s personal experiences of IPV. Many practitioners seemed to feel helpless or inadequate when offering care to abused women. This was particularly true among staff at lower levels of hospital care, particularly where they had scarce resources, lack of local support services and limited access to training on IPV. Studies from other fields of health care also reported how some providers find offering emotional care to patients more difficult than any other aspects of clinical care [26,48]. The medicalised approach focuses mainly on the physical aspects of abuse and cannot help resolve women’s problem in the long term.

The feelings of inability or lack of self efficacy form an important part of the whole debate about what are realistic expectations for medical staff in terms of what they can do when addressing violence issues. In our study, providers’ sense of lack of self-efficacy is strongly bound up in the expectations of their professional role and what the meaning of successful patients’ outcome is. It seems that for many health providers the inability to resolve an abuse case – with a woman either reporting or leaving the husband – led to a feeling of inadequacy in their job.

On the other hand, our findings show that some doctors and nurses who would go beyond the limited medical role and argue they could provide more comprehensive care to women, taking a more proactive “advocate” role, offering advice and options of referrals and help channel women to additional care. This is an image that seems to be perceived primarily at tertiary care level, where they feel their role is to help women solve their problem not only by reassuring and calming them, but also by offering advice on support services. At district level, providers seemed to see their role as primarily being medical. Sometimes, this was due to unavailability of services on-site and locally, rather than unwillingness to help women. In general though, there seems to be a widespread uncertainty among providers about what their role should include. More sensitisation can help health care workers feel less inadequate.

The systematic and documented data collection and analysis employed in the study helped ensure that the process is auditable and replicable. Respondent validation was also used, which involved feeding back the research evidence to the research participants to confirm the findings. However, the study has several limitations. Firstly, it is based on a relatively small sample of practitioners and like all qualitative work is context-specific. However, it still sheds light on the dilemmas and frustrations that many health providers may face when responding to IPV, which could be addressed in training programmes and medical curricula to improve health responses to IPV. Secondly, it does not bring the perspective of the female clients directly, as they could not be interviewed because of ethical issues. However, the extensive literature research done to conceptualise this study does focus on women’s issues in order to voice their needs when implementing an integrated response.

## Conclusion

Although many providers disagree with violence as a means to solve marital conflict and label it unfair, being part of a culture that ignores violence against women seems to affect the views of some health care professionals, and therefore it falls down the list of priorities in a busy A&E context.

Recognizing the impacts that providers views and attitudes on IPV and on their professional role can have on the quality of IPV services not only helps us comprehend why they operate in certain ways, but also make us realize the constraints that exist in promoting empathetic health care provision and which need to be addressed*.* More awareness training and sensitisation can help them feel less inadequate, especially if courses focus on women’s needs and strengths, how health providers can validate these and contribute to a longer term process of change for survivors of violence. A supportive, well resourced environment in terms of legal, counselling and police support services undoubtedly influences a health worker’s perceived ability to respond to violence. Clear guidance on how to record history of abuse, ask questions sensitively and validate experiences is also important together with training on good communication skills such as listening and being empathetic.

## Competing interests

The authors declared that they have no competing interests.

## Authors’ contributions

MC conceived the project, coordinated and conducted the study, analysed the data, and drafted the manuscript. SM helped in the conception of the study, contributed to data analysis and drafting of the manuscript. SHA participated in data analysis and help draft the manuscript. RS helped in the supervision of data collection, and contributed to data analysis. CW helped in the conceptualized the idea for the manuscript and the draft of the manuscript. All authors read and approved the final manuscript.

## Pre-publication history

The pre-publication history for this paper can be accessed here:

http://www.biomedcentral.com/1472-6963/13/65/prepub
